# Expression of *TCN1* in Blood is Negatively Associated with Verbal Declarative Memory Performance

**DOI:** 10.1038/s41598-018-30898-5

**Published:** 2018-08-23

**Authors:** Ibrahim A. Akkouh, Torill Ueland, Ole A. Andreassen, Hans-Richard Brattbakk, Vidar M. Steen, Timothy Hughes, Srdjan Djurovic

**Affiliations:** 1 NORMENT, KG Jebsen Centre for Psychosis Research, Institute of Clinical Medicine, University of Oslo, Oslo, Norway; 2 Division of Mental Health and Addiction, Oslo University Hospital, Oslo, Norway; 3 Department of Psychology, University of Oslo, Oslo, Norway; 4 NORMENT, K.G. Jebsen Centre for Psychosis Research, Department of Clinical Science, University of Bergen, Bergen, Norway; 5 Dr. E. Martens Research Group for Biological Psychiatry, Department of Medical Genetics, Haukeland University Hospital, Bergen, Norway; 6 Department of Medical Genetics, Oslo University Hospital, Oslo, Norway

## Abstract

Memory is indispensable for normal cognitive functioning, and the ability to store and retrieve information is central to mental health and disease. The molecular mechanisms underlying complex memory functions are largely unknown, but multiple genome-wide association studies suggest that gene regulation may play a role in memory dysfunction. We performed a global gene expression analysis using a large and balanced case-control sample (n = 754) consisting of healthy controls and schizophrenia and bipolar disorder patients. Our aim was to discover genes that are differentially expressed in relation to memory performance. Gene expression in blood was measured using Illumina HumanHT-12 v4 Expression BeadChip and memory performance was assessed with the updated California Verbal Learning Test (CVLT-II). We found that elevated expression of the vitamin B12-related gene *TCN1* (haptocorrin) was significantly associated with poorer memory performance after correcting for multiple testing (*β* = −1.50, p = 3.75e-08). This finding was validated by quantitative real-time PCR and followed up with additional analyses adjusting for confounding variables. We also attempted to replicate the finding in an independent case-control sample (n = 578). The relationship between *TCN1* expression and memory impairment was comparable to that of important determinants of memory function such as age and sex, suggesting that *TCN1* could be a clinically relevant marker of memory performance. Thus, we identify *TCN1* as a novel genetic finding associated with poor memory function. This finding may have important implications for the diagnosis and treatment of vitamin B12-related conditions.

## Introduction

The capacity to form short and long-term memories is indispensable for the normal functioning of our cognitive abilities, and the central role played by memory impairment in the development of severe psychiatric disorders is well established^1,2^. For these reasons, the molecular underpinnings of memory formation and retrieval have been studied extensively, and basic molecular mechanisms underlying memory function have been identified^3,4^. However, most of what we know about the molecular biology of memory is confined to relatively simple memory performances involving simple sensory stimuli like tone, touch, or shock in invertebrates and non-human vertebrates^3^. The molecular basis of more complex forms of memory, such as human declarative memory, which involves the conscious recollection of factual information or previous experiences, remains largely unknown^5^.

Genome-wide association studies (GWAS) have discovered multiple genetic variants associated with memory performance^6–8^. An interesting aspect of these studies is the large proportion of associated loci that are located in non-coding regions of the genome (intergenic or intronic). This indicates that the genetic architecture of complex memory functions primarily involves variants that exert their effects through regulating gene expression rather than altering protein structure and function^6^. Studying the transcriptional profiles of declarative memory performance may thus provide useful insights into the initial links between statistical genetic associations and cellular pathways. Moreover, such insights may help to elucidate the complex molecular mechanisms underlying severe psychiatric illnesses like schizophrenia (SCH) and bipolar disorder (BD), since both disorders are strongly characterized by cognitive dysfunction in general and memory impairment in particular^1,2^.

In the present study, we explored the genome-wide transcriptional basis of verbal declarative memory. This kind of declarative memory involves the verbal recall of facts and events and is among the most affected cognitive domains in SCH and BD^9–13^. Previous studies examining the expressional profile of memory have generally been hypothesis-driven, limiting themselves to investigating the regulatory patterns of candidate genes instead of global expression patterns^14–17^. Additionally, most of these studies examined the association between gene expression and general cognitive impairment rather than specifically measuring verbal declarative memory. One exception is a recent paper by Zheutlin *et al*., in which genome-wide peripheral expression with regard to verbal memory was assessed^18^. However, this study was limited by a relatively small sample size (n = 190) and a lack of adjustment for critical variables, such as medication use, which is known to influence both gene expression and cognitive performance^19–22^. Addressing these limitations may therefore increase the power to identify differentially expressed genes of smaller effect sizes, as well as help to distinguish the real effects of altered expression levels from those of secondary factors.

The aim of our study was three-fold. First, we performed a global gene expression analysis in peripheral blood using a large case-control sample (n = 754) aiming to discover novel genes that are differentially expressed in relation to memory performance. Second, we sought to replicate any significant finding in a non-overlapping sample (n = 578) drawn from the same geographical area. Finally, we followed up any discovery with biological relevance and strong statistical significance with additional analyses, adjusting for potentially confounding variables.

## Results

### Genome-wide screening of expression markers

**Figure 1:**
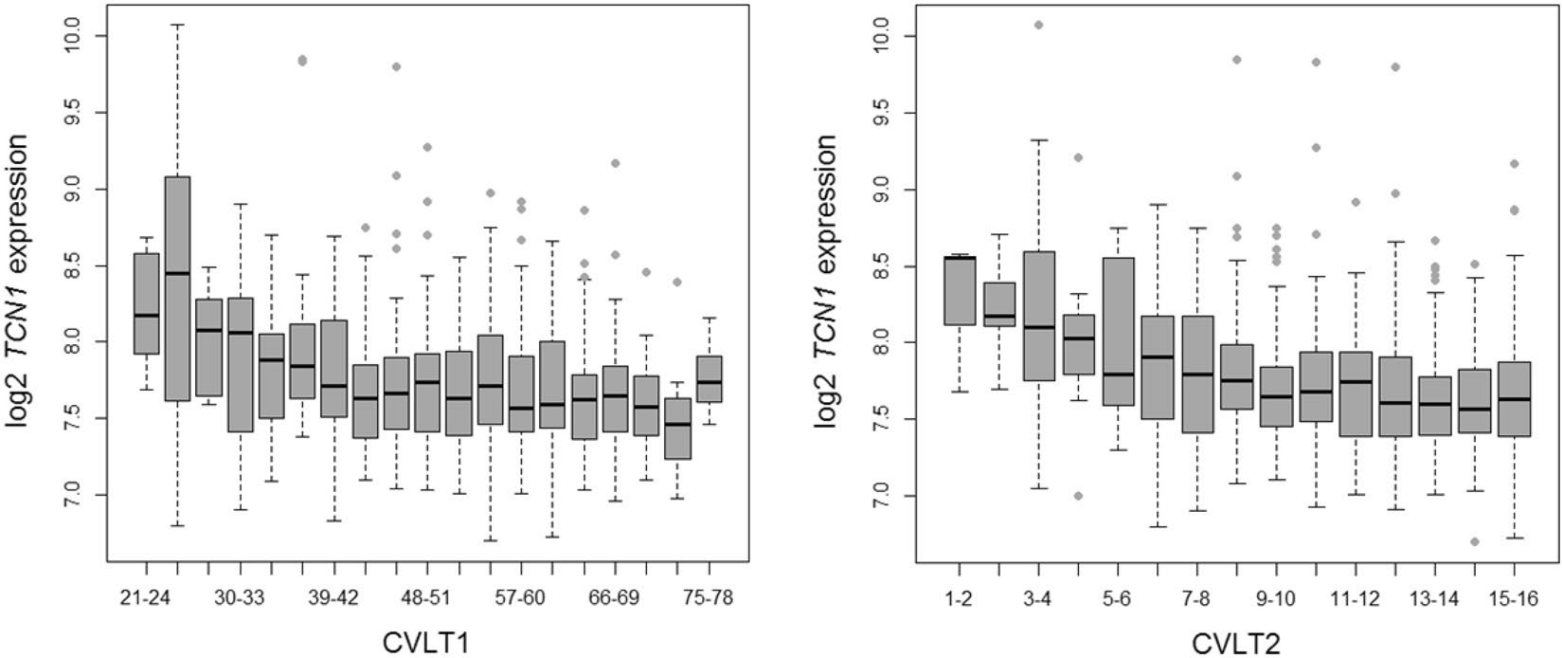
Association between *TCN1* Expression and Two Memory Measures. *TCN1* expression was negatively correlated with both CVLT1 (Pearson’s *R*: −0.220) and CVLT2 (Pearson’s *R*: −0.247). CVLT1: Verbal learning subtest. CVLT2: Long delay free recall subtest.

An initial genome-wide expression analysis was performed to identify associations between 23,476 genetic markers and two memory test scores. The verbal learning measure (CVLT1; Table 1) was significantly associated with one marker after adjusting for multiple testing (α = 1.06e-6): the *HLA-DRA* probe ILMN_2157441 (*β* = 6.57, p* = *4.90e-7, 95% CI [4.03, 9.11]). The long delay free recall measure (CVLT2; Table 2) was only significantly associated with the *TCN1* marker (*β* = −1.50, p* = *3.75e-8, 95% CI [−2.02, −0.97]). *TCN1* was also among the top ten genes associated with CVLT1 performance (*β* = −4.43, p* = *4.42e-6, 95% CI [−6.30, −2.55]). The variance in memory performance explained by *TCN1* expression was comparable in the two models (partial *R*^2^_*CVLT1*_ = 0.030; partial *R*^2^_*CVLT*2_ = 0.043). Furthermore, *TCN1* had a negative correlation with both test scores (CVLT1: Pearson’s *R* = −0.220, 95% CI [−0.290, −0.148]; CVLT2: Pearson’s *R* = −0.247, 95% CI [−0.316, −0.176]; Fig. 1). Two different markers targeting the gene *RNA18S5* were among the top ten candidates in both memory scores, but these markers did not survive correction for multiple testing (Tables 1 and 2).

**Table 1:** Top Ten Associations between Gene Expression and Verbal Learning (CVLT1).

Probe name	Gene symbol	Gene name	Gene function	*β*	95% CI	Std *β*	p-value Nominal	p-value Bonferroni	Adjusted *R*^2^
ILMN_2157441	*HLA-DRA*	Major histocompatibility complex, class II, DR alpha	Immune system, antigen presentation	6.57	4.03, 9.11	0.56	4.90e-07	0.023^*^	0.101
ILMN_2114720	*SLPI*	Secretory leukocyte peptidase inhibitor	Immune system, protection of epithelial tissues from serine proteases	−5.70	−8.01, −3.39	−0.49	1.56e-06	0.073	0.098
ILMN_3239610	*RNA18S5*	RNA, 18 S ribosomal 5	Structural ribosomal component	−2.39	−3.39, −1.40	−0.20	2.88e-06	0.135	0.097
ILMN_1703337	*RNA18S5*	RNA, 18S ribosomal 5	Structural ribosomal component	−2.64	−3.75, −1.53	−0.23	3.37e-06	0.158	0.097
ILMN_1712888	*HSPH1*	Heat shock protein family H member 1	Protein folding	7.22	4.18, 10.27	0.62	3.72e-06	0.175	0.096
ILMN_2041046	*CKS1B*	CDC28 protein kinase regulatory subunit 1B	Cell cycle regulation	12.41	7.15, 17.67	1.06	4.32e-06	0.203	0.096
ILMN_1768469	*TCN1*	Transcobalamin 1; haptocorrin	Vitamin B12 transportation and cellular uptake	−4.43	−6.30, −2.55	−0.38	4.42e-06	0.207	0.096
ILMN_1812433	*HP*	Haptoglobin	Binding of free plasma hemoglobin	−7.30	−10.52, −4.07	−0.62	1.04e-05	0.486	0.094
ILMN_1750661	*FBXW9*	F-box and WD repeat domain containing 9	Ligation of ubiquitin to proteins	−13.40	−19.44, −7.35	−1.14	1.57e-05	0.737	0.093
ILMN_3251587	*RNA*2*8S5*	RNA, 28S ribosomal 5	Structural ribosomal component	−1.52	−2.22, −0.83	−0.13	1.79e-05	0.843	0.092

Std *β*: Standardized regression coefficients.

*Bonferroni-adjusted p-value < 0.05.

**Table 2:** Top Ten Associations between Gene Expression and Long Delay Free Recall (CVLT2).

Probe name	Gene symbol	Gene name	Gene function	*β*	95% CI	Std *β*	p-value Nominal	p-value Bonferroni	Adjusted *R*^*2*^
ILMN_1768469	*TCN1*	Transcobalamin 1; haptocorrin	Vitamin B12 transportation and cellular uptake	−1.50	−2.02, −0.97	−0.46	3.75e-08	0.0018^*^	0.095
ILMN_1703337	*RNA18S5*	RNA, 18S ribosomal 5	Structural ribosomal component	−0.74	−1.05, −0.43	−0.23	4.05e-06	0.190	0.083
ILMN_1693192	*PI3*	Peptidase inhibitor 3	Protection againts Gram-positive and Gram-negative bacteria	−0.63	−0.89, −0.36	−0.19	4.72e-06	0.221	0.082
ILMN_1712522	*CEACAM6*	Carcinoembryonic antigen related cell adhesion molecule 6	Cell adhesion	−1.23	−1.77, −0.69	−0.38	8.34e-06	0.392	0.081
ILMN_3239610	*RNA18S5*	RNA, 18S ribosomal 5	Structural ribosomal component	−0.64	−0.92, −0.36	−0.20	8.76e-06	0.411	0.081
ILMN_1761941	*FAM198B*	Family with sequence similarity 198 member B	Subfamily of the GASK (Golgi-Associated Kinase) family of secretory kinases	−1.56	−2.25, −0.87	−0.47	1.10e-05	0.516	0.080
ILMN_1692223	*LCN2*	Lipocalin 2	Transportation of small hydrophobic molecules	−0.76	−1.098, −0.42	−0.23	1.22e-05	0.571	0.080
ILMN_1751607	*FOSB*	FosB proto-oncogene, AP-1 transcription factor subunit	Regulation of cell proliferation, differentiation, and transformation	3.38	1.86, 4.90	1.03	1.38e-05	0.649	0.079
ILMN_2114720	*SLPI*	Secretory leukocyte peptidase inhibitor	Immune system, protection of epithelial tissues from serine proteases	−1.46	−2.11, −0.80	−0.44	1.42e-05	0.666	0.079
ILMN_3249578	*RNA28S5*	RNA, 18S ribosomal 5	Structural ribosomal component	−0.56	−0.82, −0.31	−0.17	1.50e-05	0.703	0.079

Std *β*: Standardized regression coefficients.

^*^Bonferroni-adjusted p-value < 0.05.

### Differential expression between diagnostic groups

**Figure 2:**
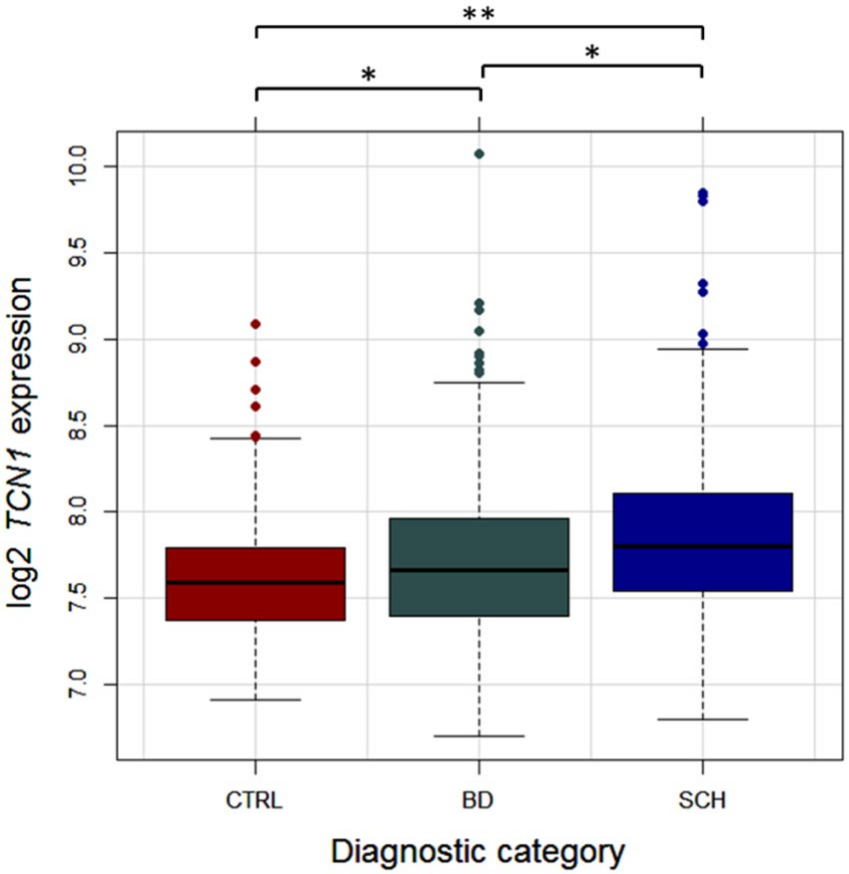
Pairwise Comparisons of *TCN1* Expression across Diagnostic Categories. Expression of *TCN1* (F(2, 751) = 20.87, p = 1.5e-9) was significantly different between diagnostic groups. BD: bipolar disorder, CTRL: healthy controls, SCH: schizophrenia. ^*^p > 0.05. ^**^p < 0.001.

We found a significantly different expression across diagnostic groups for both *TCN1* (*F*(2,751) = 20.87, p = 1.5e-9, *R*^2^ = 0.050) and *HLA-DRA* (*F*(2,751) = 26.50, p = 7.54e-12, *R*^2^ = 0.063). *HLA-DRA* had significantly reduced expression in the BD (mean* = *12.67, sd = 0.32, p = 2.14e-9) and SCH (mean* = *12.67, sd = 0.33, p = 4.62e-10) groups compared to the CTRL group (mean* = *12.86, sd = 0.31), but no significant difference was found between the patient groups (p = 0.99; see Supplementary Figure S1), suggesting that the underlying immune-related mechanisms are partly similar in the two disorders. Expression of *TCN1* was significantly elevated in SCH (mean* = *7.87, sd = 0.48) compared to both BD (mean* = *7.73, sd = 0.48) and CTRL (mean* = *7.61, sd = 0.37). All group differences met the Bonferroni-corrected significance threshold for pairwise comparisons (SCH-CTRL: p = 7.6e-10; SCH-BD: p* = *1.21e-3; BD-CTRL: p* = *0.019; Fig. 2).

### *TCN1* association

To exclude the potentially confounding effects of other variables, we performed a final regression analysis in which we controlled for medication use, diagnosis, education, and vitamin B12 serum levels in addition to age and sex. After adjusting for these confounders, the *TCN1* marker was still nominally significantly associated with both verbal learning (*β* = −3.65, p = 2.60e-3, 95% CI [−6.01, −1.28]) and long delay free recall (*β* = −1.24, p = 4.05e-4, 95% CI [−1.92, −0.56]). Both the effect size of, and the variance explained by, *TCN1* expression were comparable to that of age, sex, education, and psychiatric disease, all of which are variables that are known to influence memory function (Table 3).

**Table 3:** Regression Coefficients of Predictor Variables in Final CVLT Models.

Predictor	CVLT1				CVLT2			
	*β*	std *β*	Partial *R*^*2*^	p-value	*β*	std *β*	Partial *R*^*2*^	p-value
*TCN1*	−3.65	−1.67	0.024	0.0026^*^	−1.24	−0.57	0.033	4.05e-4^*^
Age	−0.27	−2.83	0.058	2.30e-6^*^	−0.058	−0.61	0.033	3.99e-4^*^
Sex (male)	−4.23	−2.11	0.036	2.07e-4^*^	−0.97	−0.48	0.023	0.0031^*^
Education (years)	0.75	2.10	0.039	5.09e-4^*^	0.19	0.54	0.030	0.0018^*^
Vitamin B12	−0.0074	−0.96	2.0e-3	0.088	−0.0014	−0.18	2.4e-4	0.27
Medication use (yes)	−1.68	−0.83	2.2e-3	0.36	0.26	0.13	6.5e-4	0.62
Psychiatric illness (yes)	−3.43	−1.62	8.1e-3	0.082	−1.66	−0.78	0.022	0.0039^*^

Standardized *β* values were calculated after converting all predictor variables to z-scores. CVLT1: Verbal learning score. CVLT2: Long delay free recall score. Std *β*: Standardized beta coefficients. *p < 0.01.

The relationship between *TCN1* expression and three subcategories of declarative memory was investigated in order to identify which memory process is the most affected by *TCN1* expression. The standardized effect size and the p-value of recognition memory were almost identical to those of verbal learning (CVLT1), indicating that recognition memory along with long-term memory (CVLT2) are the memory domains that are most affected by *TCN1* expression (see Supplementary Table S1).

The same two models (initial and final) were used to examine the association between *TCN1* expression and verbal learning in the replication sample using HVLT performances converted to z-scores as the memory measure. Despite the change in memory metric, a significant negative correlation was found when age and sex were adjusted for (*β* = −0.0031, p = 0.0015, 95% CI [−0.0050, −0.0012]). In the final HVLT analysis with adjustment for multiple variables, a negative effect of *TCN1* expression with a p-value just above the significance level was found (*β* = −0.0018, p = 0.062, 95% CI [−3.64e-3, 8.64e-5]). Although the effect sizes in the CVLT and HVLT models were not comparable in magnitude, the direction was the same for both tests and the variance explained by the two models was similar (see Supplementary Table S2).

### Verification of *TCN1* microarray measurements

We verified the microarray-based *TCN1* expression data by comparing them to qPCR measurements. qPCR is an orthogonal technology which is considered the golden standard for differential expression profiling. The two methods showed a good overall concordance (n = 82, Pearson’s *R* = 0.816, 95% CI: 0.73, 0.88; see Supplementary Figure S2), and the strength of correlation was comparable to that of previous studies^23–25^.

### Expression Quantitative Trait Loci (eQTL) analysis

We examined 127 *TCN1*-related SNPs for associations with *TCN1* expression and found that 6 were nominally significant at the 0.05 significance level. However, none of these SNPs survived correction for multiple testing (see Supplementary Table S3, Supplementary Figures S3, and S4).

## Discussion

In the present study, we performed a genome-wide expression analysis to examine the relationship between gene expression in peripheral blood and verbal declarative memory, using the verbal learning (CVLT1) and long delay free recall (CVLT2) measures from the updated California Verbal Learning Test. We identified two genes that were significantly related to memory performance. The first gene, *HLA-DRA*, was positively correlated with the verbal learning measure. *HLA-DRA* (Major Histocompatibility Complex, Class II, DR Alpha) is located within the Major Histocompatibility Complex (MHC) locus on chromosome 6 and plays an important role in immune regulation by presenting antigenic peptides to T cells^26^. The MHC region is one of the strongest and most significant susceptibility loci for schizophrenia^27–29^, and the locus has also been implicated in declarative memory impairment^30^. The second identified gene was *TCN1*, which was significantly associated with long delay free recall performance and among the top ten genetic markers associated with verbal learning. *TCN1* expression was negatively correlated with both measures. This inverse correlation is consistent with the fact that *TCN1* is able to bind circulating vitamin B12 but unable to mediate cellular uptake, and with the well-known effect of vitamin B12 deficiency on memory performance^31^.

### *TCN1*’s Role in Cobalamin Transportation

**Figure 3:**
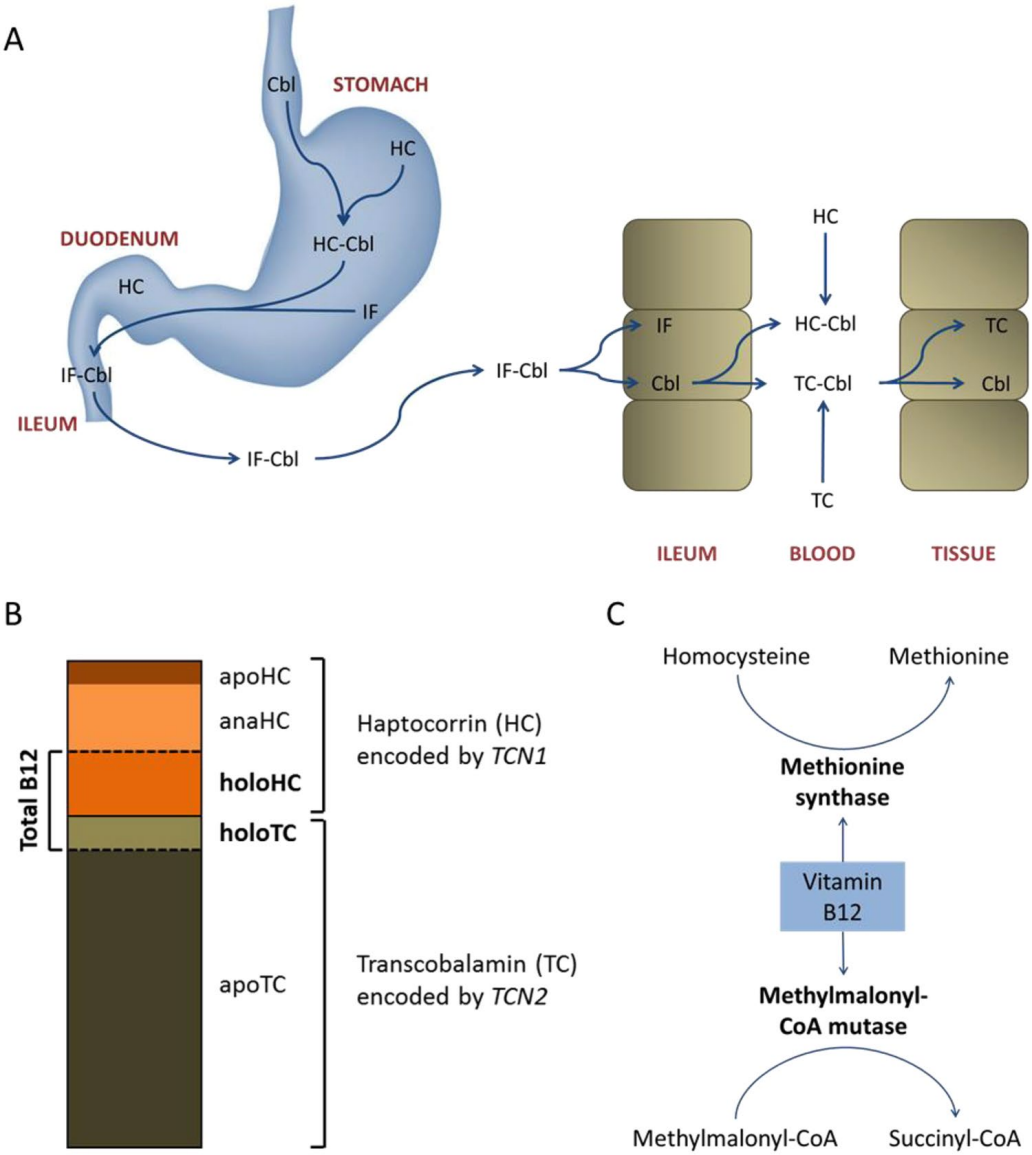
Cobalamin Transportation, Binding Proteins, and Metabolic Pathways. (**A**) The transportation of cobalamin (Cbl) through the gastrointestinal tract and its subsequent release into the blood stream is a multistep process requiring the concerted action of three proteins: intrinsic factor (IF), haptocorrin (HC), and transcobalamin (TC). After Cbl has been released from food, it is first bound by HC secreted mainly from salivary glands but also from parietal cells in the stomach^32^. HC forms a complex with Cbl and carries it through the stomach while protecting it from hydrolysis in the acidic environment. In the duodenum, HC is cleaved off from the complex by pancreatic enzymes and IF, the second Cbl-binding protein, transports Cbl to the terminal ileum. Here, Cbl is absorbed by intestinal cells through receptor-mediated endocytosis and subsequently released into the blood stream^37,75^. In the blood, ~80% of Cbl is bound to HC (holoHC), while the remainder is bound by TC (holoTC). Only the smaller holoTC fraction is available for cellular uptake. (**B**) Plasma cobalamin and its binding proteins: HC is almost fully saturated with Cbl (holoHC) and its inactive derivatives, so-called cobalamin analogues (anaHC). Only ~10% of plasma HC exists as freely circulating HC (apoHC). Adapted from Nexo *et al*.^59^. (**C**) Cobalamin is an essential cofactor for the enzymes methionine synthase and methylmalonyl-CoA mutase. The enzymatic reactions carried out by these enzymes form part of complex pathways required for the biosynthesis of chemical compounds that are indispensable for the proper functioning of the central nervous system. When cellular B12 levels are insufficient, homocysteine and methylmalonic acid (MMA), the CoA-free form of methylmalonyl-CoA, accumulate in the cell and subsequently in the blood stream. Serum concentrations of homocysteine and MMA are negatively correlated with circulating holoTC^59^.

*TCN1* (transcobalamin 1; the protein is more commonly referred to as haptocorrin) encodes one of three vitamin B12-binding proteins and is expressed in multiple tissues and secretions^32^. An intricate multistep process is required for the transportation of vitamin B12 (hereafter referred to as cobalamin) through the gastrointestinal tract and the subsequent uptake into the various tissues of the body^33^ (Fig. 3A). In the blood stream, cobalamin is bound to either haptocorrin (HC) or transcobalamin (TC; encoded by the gene *TCN*2) (Fig. 3B). HC is almost fully saturated with cobalamin and binds ~80% of the vitamin with very high affinity, whereas the remainder is bound by TC (holoTC) with higher specificity^32,34,35^. However, only the smaller fraction of TC-bound cobalamin, so-called active cobalamin, is available for cellular uptake^36^. The physiological function of circulating HC is not fully understood, but its distinctive ability to bind enzymatically inactive forms of the vitamin with equally high efficiency suggests a role in cobalamin storage and the scavenging of inhibitory cobalamin derivatives^34,37^.

### Cobalamin Metabolism and Memory Performance

The central role of cobalamin in important metabolic pathways is well established^38,39^. Once taken up by the body’s cells, cobalamin acts as an essential cofactor for two enzymes: methionine synthase, which catalyzes the synthesis of methionine from homocysteine in the cytosol; and methylmalonyl-CoA mutase, which catalyzes the mitochondrial conversion of methylmalonyl-CoA to succinyl-CoA^33^ (Fig. 3C). These enzymatic reactions are necessary for the biosynthesis of several molecules that are indispensable for the normal functioning of the central nervous system, including nucleotides, myelin phospholipids, and neurotransmitters^31,40,41^. The involvement of cobalamin in these biosynthetic pathways may therefore partly explain the diversity of clinical and neurological manifestations of cobalamin-related deficiencies. Of particular relevance, reduced cellular uptake of cobalamin is associated with psychotic symptoms, depression, mania, cognitive impairment, and memory loss^31,40–44^. The association between cobalamin deficiency and memory loss is especially interesting, as individuals with low-normal cobalamin levels have significantly lower hippocampal microstructure integrity compared to individuals in the normal-high range^45^.

Thus, *TCN1*’s role in cobalamin metabolism and the known effect of cobalamin deficiency on memory function provide a plausible and clinically relevant biological mechanism for the inverse correlation between *TCN1* expression and memory performance in a cobalamin-sufficient context (the majority of the subjects in the discovery sample had sufficient cobalamin levels; see Supplementary Table S6). When HC levels in plasma are high, a larger fraction of circulating cobalamin is bound by HC due to its superior affinity in comparison to TC. Consequently, cellular absorption of active cobalamin is reduced, leading to the disruption of metabolic pathways that involve the production of essential chemical compounds, negatively affecting memory function. Interestingly, the pattern of differential *TCN1* expression with respect to diagnostic status is also consistent with the proposed mechanism. In our sample, expression of *TCN1* gradually increased going from healthy controls to BP patients to SCH patients (Fig. 2). This pattern is in accordance with the well-established finding that cognitive deficits are generally more prominent in schizophrenia than in bipolar disorder^46–48^. Our results suggest that transcriptional dysregulation of *TCN1* may be a contributing factor to the cognitive impairments associated with severe mental disorders.

### *TCN1* an Important Marker of Memory Performance

Confounding factors are a consistent source of uncertainty in clinical studies. An important strength of our methodological design was the adjustment for critical variables that are either known to influence both gene expression and cognitive performance, such as age, sex, and medication use^49–55^; or are potential confounders due to their causal associations with either of the predictor or response variables, such as educational attainment, mental disorders, and cobalamin serum levels^56,57^. By controlling for these factors, we were able to largely exclude the possible effects of secondary contributors. Considering the significant *TCN1* correlation in our final analysis where we control for mental disease (Table 3), it does not seem that diagnostic status is the main driver of the negative effect of *TCN1* expression on memory function. This is also indicated by the fact that we obtained similar results when performing stratified analyses in which each diagnostic subgroup was examined separately (see Supplementary Table S4 and Supplementary Figure S5). Furthermore, the magnitude of the *TCN1* effect on memory performance was comparable to that of sex, age, education, and mental disease (Table 3), suggesting that *TCN1* is an important and clinically relevant marker of memory-related functions.

### Measurement of Cobalamin in Serum

It is well established that only holoTC, the smaller fraction of circulating cobalamin bound to TC, is available for cellular utility. Consequently, it was suggested three decades ago that serum levels of holoTC could serve as an optimal marker for cobalamin deficiency^58^. At the time, the uncertainty in holoTC measurements was too large to be useful in clinical practice^59^. Subsequent research deploying more sensitive methods, however, has confirmed the soundness of this suggestion^59–61^. In addition, studies examining the relationship between total serum cobalamin and memory impairment have yielded conflicting results, indirectly confirming the clinical and diagnostic advantage of utilizing holoTC^62–65^. Other indicators of cobalamin status include homocysteine and methylmalonic acid (MMA), both of which are essential constituents of cobalamin-related metabolic pathways. Although measurement of these metabolites appears to be as sensitive as holoTC, a drawback of both of them is poor specificity^59,66^. Despite the apparent advantages of measuring holoTC, there seems to be a general reluctance to begin using the test in both clinical and research practice. Our finding that *TCN1* has a negative effect in individuals that are cobalamin sufficient further strengthens the case for measuring holoTC rather than total cobalamin in both clinical and research applications.

### Blood as a Relevant Tissue for Neurocognitive Function and Disorders

Since gene expression is tissue-specific, it is generally assumed that only brain tissue is directly relevant for the transcriptional examination of neurocognitive function and mental disease. However, researchers are often compelled to use peripheral gene expression as a surrogate for brain expression due to the limited accessibility and the ethical considerations involved in acquiring human brain samples. This is accepted practice because whole blood shares significant transcriptional similarities with multiple tissues in the central nervous system^67^. This was in fact the logic behind the design of the present study, but based on our findings it is interesting to speculate whether peripheral blood itself could be a partly relevant tissue for expressional modelling of cognitive functions and psychiatric diseases. Both *HLA-DRA* and *TCN1* are highly expressed in whole blood, while the mRNA levels in brain are very low or non-existent (www.gtexportal.org), suggesting that the use of blood in addition to brain tissue could be important in order to achieve a complete transcriptomic profiling of brain disorders. Given that immune-related loci are among the most significant and persistent findings in schizophrenia^28^, it appears that whole blood is a relevant tissue when exploring the expressional status of at least a subset of disease-linked genes.

### Limitations of the Present Study

The clinical interpretation of our findings relies on the assumption that there is a positive correlation between serum mRNA levels and total serum protein concentrations of a given gene. Although this assumption is generally accepted, it does not always hold. In the case of *TCN1*, results should be interpreted cautiously even if the assumption holds since only the smaller fraction of circulating HC which is bound to cobalamin (holoHC; Fig. 3C) can influence cobalamin metabolism. For this reason, further studies aimed at holoTC and holoHC measurements and association with memory performance are highly warranted.

In the replication analysis of *TCN1*, we used HVLT as the verbal learning measure for the majority of study participants. Although the verbal learning measure of HVLT has been shown to be significantly related to the corresponding measure in CVLT^68^ with a relatively high correlation (*r* = 0.74), comparisons between the two tests should be made with caution. This is because the list of words in HVLT is shorter compared to CVLT, and the test may therefore be less sensitive to detect memory decline than CVLT^68^. The fact that we obtained positive replication results despite this limitation could be an indication of the robustness of our primary *TCN1* finding.

## Conclusions

In summary, the present study identifies *TCN1* (haptocorrin) as a novel genetic finding associated with poor declarative memory function. The effect size of *TCN1* expression on memory performance is comparable to that of age, sex, and mental illness, all of which are known determinants of memory function. This implies that the role played by *TCN1* could be of clinical importance and relevance. The present findings also suggest that the transcriptional status of this gene could have a diagnostic potential in the assessment of conditions related to cobalamin metabolism. Furthermore, the expression pattern of *TCN1* across diagnostic categories suggests that the cognitive impairments associated with SCH and BD may partly be attributed to changes in *TCN1* expression levels. Finally, our results underscore the clinical importance of using holoTC as a biomarker for cobalamin status rather than total cobalamin.

## Methods

### Sample characteristics

The discovery sample (n = 754) consisted of 229 healthy controls (CTRL), 234 bipolar disorder patients (BD; 152 type I, 66 type II, 16 not otherwise specified), and 291 schizophrenia spectrum patients (SCH; 212 schizophrenia, 56 schizoaffective, 23 schizophreniform; see Supplementary Table S6). The replication sample (n = 578) consisted of 316 CTRL, 67 BD patients (62 type I and 5 not otherwise specified), and 195 SCH patients (142 schizophrenia, 35 schizoaffective, 18 schizophreniform; see Supplementary Table S7). All patients were diagnosed according to the Structured Clinical Interview for DSM-IV Axis I disorders (SCID-I)^69^. Individuals in the CTRL group were excluded if they or their close relatives had a lifetime history of severe psychiatric disorder. All participants gave written informed consent and the study was approved by the Norwegian Regional Committee for Medical Research Ethics and the Norwegian Data Inspectorate. All procedures and methods were carried out in accordance with relevant guidelines and regulations. The recruitment procedure and clinical evaluation for both samples are described in detail in previous reports^70,71^.

### Neurocognitive assessment

Neurocognitive assessment of all study participants was carried out by psychologists trained in standardized neuropsychological testing. The three-hour test battery was administered in a fixed order with two breaks, and included the updated California Verbal Memory Test (CVLT-II) with subscore measures of verbal learning (CVLT1) and long delay free recall (CVLT2)^72^. Participants were read a list of 16 words in fixed order over five learning trials. After each trial, they were asked to recall, in any order, as many words as they could remember (verbal learning). They were also asked to recall the words after 20 minutes (long delay free recall). The performance metrics used in this study were the sum of scores across the five learning trials (CVLT1) and the long delay measure (CVLT2). The majority of subjects in the replication sample (n = 465) were not assessed with CVLT-II, but with the highly similar Hopkins Verbal Learning Test (HVLT)^73^. In this test, participants were read a list of 12 words over three learning trials. The sum of scores was used as the verbal learning performance metric in the replication analyses.

### RNA microarray analysis and quality control

Blood samples were collected in Tempus Blood RNA Tubes (Life Technologies Corporation). Total RNA was extracted with the TEMPUS 12-Port RNA Isolation Kit (Applied Biosystems) and ABI PRISM 6100 Nucleic Acid PrepStation (Applied Biosystems) according to manufacturer’s protocol. Global gene expression analyses were performed with Illumina HumanHT-12 v4 Expression BeadChip (Illumina, Inc.) consisting of ~47,000 probes. Multidimensional scaling and hierarchical clustering were used for regular quality control, including sample quality measurements and removal of outliers, as well as removal of multiple batch effects (RNA extraction batch, RNA extraction method, DNase treatment batch, cRNA labelling batch, and chip hybridization). This was followed by log2-transformation. More details on microarray preprocessing and quality control are provided in the online supplementary material. Probes showing zero expression in more than 90% of the samples were ignored, leaving 23,476 markers left for examination. All genome-wide expression analyses were performed on the batch-adjusted log2-transformed data.

### Validation of microarray data with qPCR

Gene expression data from the microarray measurement of *TCN1* were validated with quantitative real-time PCR (qPCR). From the discovery sample, 94 individuals representing all diagnostic categories and spanning the full range of microarray expression levels were picked out for validation. High-Capacity cDNA Reverse Transcription Kit (Life Technologies Corporation) was used for reverse transcription of 1 µg RNA. The qPCR reaction was performed on 5 ng cDNA using *TCN1* TaqMan Gene Expression Assay (Hs01055542_m1; Life Technologies Corporation) as target. All samples were run in four replicates. 12 individuals were excluded due to poor cDNA quality. The results were analyzed with qbase+ software (Biogazelle, Zwijnaarde, Belgium) using the comparative cycling threshold (ΔΔCt) method^74^ with *GUSB* (Hs00939627_m1) as endogenous control and a positive cDNA control sample as reference. The qPCR measures of *TCN1* expression were regressed against the log2 microarray *TCN1* gene expression values using a linear model in R 3.4.1.

### Expression Quantitative Trait Loci (eQTL) enalysis

Subjects in the discovery sample were genotyped at Expression Analysis Inc. (Durham, USA) using the Affymetrix Genome-Wide Human SNP Array 6.0 (Affymetrix Inc., Santa Clara, CA, USA). Samples were excluded if they were low-yield (call rate below 97%); if they were duplicates of other samples included in the study; if they their sex as determined by X chromosome marker homozygosity was different from their reported sex; or if they were calculated to have other ancestry than European. All SNPs located within 20 kb upstream and downstream of the *TCN1* gene were examined for associations with *TCN1* expression. In total, 127 SNPs were investigated in 577 subjects.

### Statistical analyses

All patients and controls in the discovery sample (n = 754) were first tested for associations between genome-wide expression levels and memory performance. In the initial screening, both verbal learning (CVLT1) and long delay free recall (CVLT2) scores were examined for associations with 23,476 genetic markers in a multiple linear regression model including only age and sex as covariates. Probes with a p-value below the Bonferroni-corrected critical level of α = 0.05/(2 × 23,476) = 1.06e-6 were considered significant. One-way analysis of variance (ANOVA) was then used to examine whether the significant probes were differentially expressed across diagnostic categories. To find which specific diagnostic groups were significantly different from each other, the ANOVA analyses were followed by pairwise comparisons using Tukey-Kramer’s post-hoc test.

Since *TCN1* was among the top ten genes associated with CVLT1 performance and the only gene significantly correlated with the CVLT2 measure, as well as being a biologically and clinically interesting candidate, we explored the effect of potential confounders on the *TCN1* association. We performed a final multiple linear regression analysis in which we adjusted for age, sex, diagnosis, medication (categorized as yes or no), and education (total number of years). As *TCN1* is known to play an important role in the transportation and uptake of vitamin B12, we also controlled for total B12 serum levels. We did not adjust for multiple testing in this final analysis. Normality of the variables was checked with the *plot()* function in R 3.4.1.

To determine which memory process is most affected by *TCN1* expression, we examine the relationship between *TCN1* expression and three subcategories of declarative memory: working memory, as measured by the first learning trial of CVLT; memory consolidation (or retention rate), as calculated by dividing the long delay free recall (CVLT2) score by the last learning trial; and recognition memory, which is a separate measure within the CVLT test battery. We adjusted for age and sex in these regression analyses.

The association between *TCN1* and verbal learning was further examined in the independent replication sample (n = 578) drawn from the same geographical region. The majority of these subjects (n = 465) where not assessed with CVLT but with the highly similar Hopkins Verbal Learning Test (HVLT). Thus, both HVLT and CVLT were used as the verbal learning measure after converting both test values into z-scores. The same covariates as in the main analysis were included in the replication test. All statistical analyses were performed in R 3.4.1.

## Electronic supplementary material

Supplementary information

## Data Availability

The datasets generated and analyzed during the current study are not publicly available due to Institutional Review Board restrictions but are available from the corresponding author on reasonable request.
