# Supplementary material for: Expression of TCN1 in Blood is Negatively Associated with Verbal Declarative Memory Performance
Source: Sci Rep. 2018 Aug 23;8:12654. doi: 10.1038/s41598-018-30898-5 (PMC6107676; doi:10.1038/s41598-018-30898-5)
Supplement: Supplementary file 1 — Supplementary information [file 41598_2018_30898_MOESM1_ESM.pdf]

SUPPLEMENTARY INFORMATION

Expression of *TCN1* in Blood is Negatively Associated with Verbal Declarative Memory Performance

Ibrahim A. Akkouh[1]*, Torill Ueland[1,2,3], Ole A. Andreassen[1,2], Hans-Richard Brattbakk[4,5], Vidar M. Steen[4,5], Timothy Hughes[1,6], and Srdjan Djurovic[4,6]

* Corresponding author: ibrahim.akkouh@medisin.uio.no.

[1]NORMENT, KG Jebsen Centre for Psychosis Research, Institute of Clinical Medicine, University of Oslo, Oslo, Norway; [2]Division of Mental Health and Addiction, Oslo University Hospital, Oslo, Norway; [3]Department of Psychology, University of Oslo, Oslo, Norway; [4]NORMENT, K.G. Jebsen Centre for Psychosis Research, Department of Clinical Science, University of Bergen, Bergen, Norway; [5]Dr. E. Martens Research Group for Biological Psychiatry, Department of Medical Genetics, Haukeland University Hospital, Bergen, Norway; [6]Department of Medical Genetics, Oslo University Hospital, Oslo, Norway.

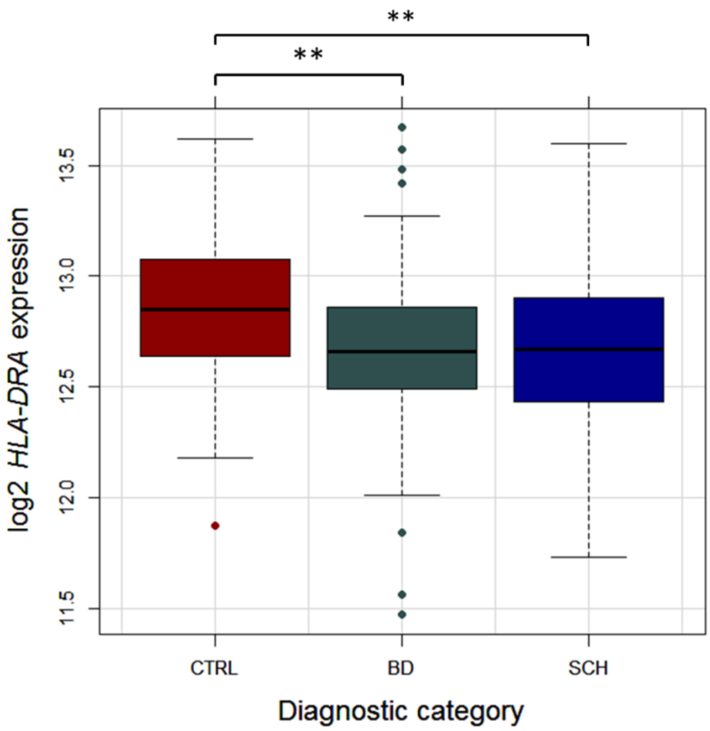

Supplementary Figure S1. Pairwise Comparisons of *HLA-DRA* Expression across Diagnostic Categories. Expression of *HLA-DRA* was significantly elevated in healthy controls compared to schizophrenia and bipolar disorder patients. BD: bipolar disorder, CTRL: healthy controls, SCH: schizophrenia. ** $p < 0.001$.

Supplementary Table S1. Association between *TCN1* Expression and Declarative Memory Subcategories

	β	95% CI	Std β	p-value
Working memory	-0.40	-0.76, -0.045	-0.19	0.028*
Memory consolidation	-0.053	-0.085, -0.022	-0.28	8.47e-04*
Recognition memory	-0.62	-0.88, -0.36	-0.38	5.97e-06*
Long-term memory (CVLT2)	-1.50	-2.02, -0.97	-0.46	3.75e-08*
Verbal learning (CVLT1)	-4.43	-6.30, -2.55	-0.38	4.42e-06*

The first learning trial of CVLT was used as a marker of working memory. Memory consolidation was calculated by dividing the long delay free recall score (CVLT2) by the last learning trial of CVLT. Recognition memory was assessed with a separate measure within the CVLT test. All analyses were adjusted for age and sex. Std β: Standardized regression coefficients. * $p < 0.05$.

Supplementary Table S2. Standardized Regression Coefficients of *TCN1* in CVLT1 and HVLT Models

			Standardized effect size	
Model	p-value	Adjusted R^2	β	95% CI
CVLT1 initial	4.42e-6*	0.096	-0.38	-0.54, -0.22
HVLT initial	0.0015*	0.045	-0.0031	-0.0050, -0.0012
CVLT1 final	0.0026*	0.20	-0.31	-0.51, -0.11
HVLT final	0.062	0.22	-0.0018	3.64e-3, 8.64e-5

Standardized effect sizes were calculated from CVLT and HVLT scores after conversion to z-scores. CVLT1: Verbal learning score. HVLT: Hopkins verbal Learning Test. Initial: Initial screening adjusted for age and sex. Final: Final analysis adjusted for multiple covariates.
* $p < 0.05$.

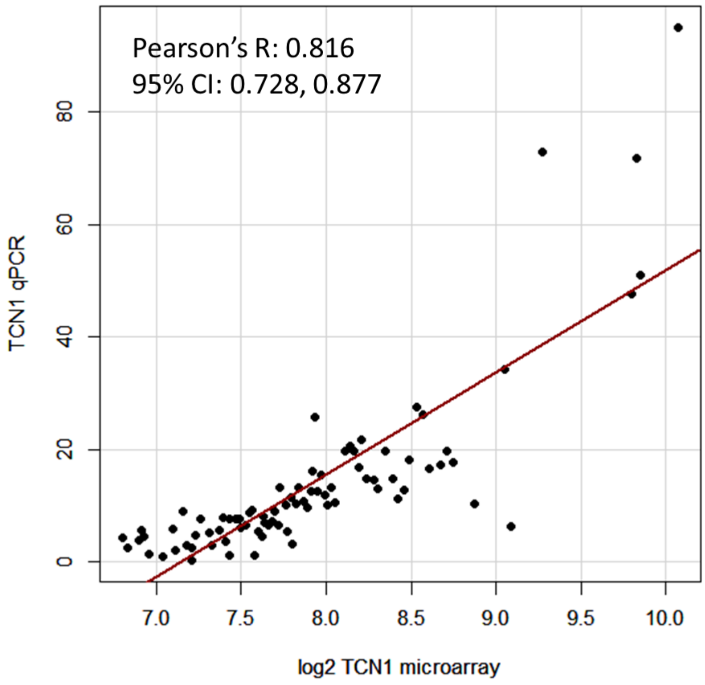

Supplementary Figure S2. Comparison between *TCN1* Expression Levels as Measured using Microarrays and Quantitative Polymerase Chain Reaction (qPCR). 82 individuals from the discovery sample representing all diagnostic categories and the full range of *TCN1* expression were picked out for qPCR validation. The two methods showed a good overall concordance (Pearson's *R*: 0.816, 95% CI: 0.728, 0.877).

Supplementary Table S3. Nominally Significant eQTLs Associated with *TCN1* Expression

Base position	Reference SNP cluster ID	t-value	p-value	FDR	β
11:59615661	rs188490025	3.29	0.0011	0.14	1.077
11:59631535	rs34528912	-3.084	0.0021	0.14	-0.081
11:59623433	rs1042613	-2.74	0.0063	0.27	-0.046
11:59619212	rs144717033	2.49	0.013	0.35	0.31
11:59606263	rs182868282	2.47	0.014	0.35	0.38
11:59634519	rs579248	-2.049	0.041	0.87	-0.018

Supplementary Figure S3. QQ-plot of P-values for 127 *TCN1*-related Single Nucleotide Polymorphisms

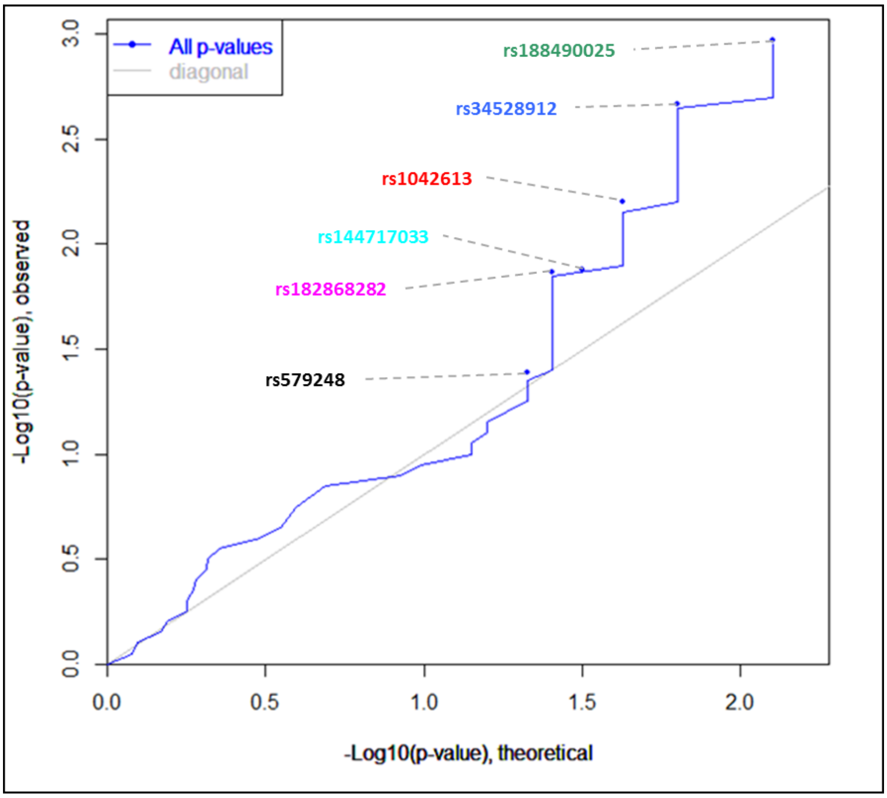

Supplementary Figure S4. Nominally Significant eQTLs Mapped to Genomic Positions in the GRCh37 Assembly

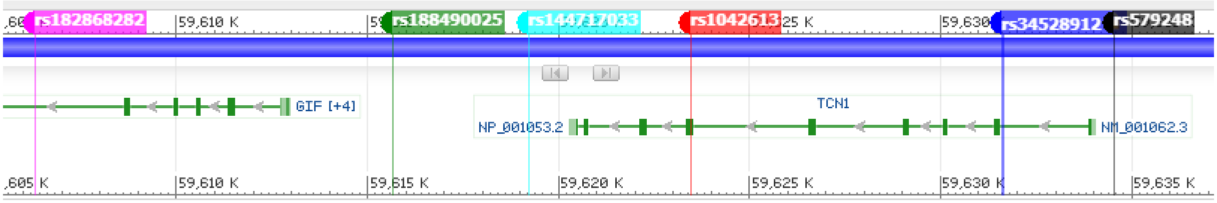

Analysis of *TCN1* Expression and Memory Stratified by Diagnostic Status

We performed additional regression analyses in which we looked at the effect of *TCN1* expression on memory performance within each diagnostic category. In these stratified analyses, we adjusted for age and sex as we did in the initial genome-wide screening. In order to keep the individual sample sizes as big as possible, and thereby avoid losing statistical power, we merged the discovery and replication samples together. Both CVLT and HVLT scores were used as the memory performance metric after transforming raw memory scores to z-scores. The association between *TCN1* expression and memory was nominally significant ($p<0.05$) in SCH and BD groups, but not in the CTRL group (Supplementary Table S4). However, the direction of the effect was as expected in all groups, i.e. a negative correlation between *TCN1* and memory (Supplementary Table S4 and Supplementary Figure S3). Moreover, the *TCN1* effect size was still comparable in magnitude to the effect sizes of age and sex, indicating that there is a real and negative effect of *TCN1* expression on memory performance, and that this effect does not seem to be explained by the psychiatric condition. The fact that the association is not significant in the CTRL group could be due to the large proportion of subjects that were tested with HVLT rather than CVLT in the CTRL group compared with the SCH and BD groups (Supplementary Table S5). As discussed in the main text, HVLT may be less sensitive to detect memory decline because of the shorter word list in HVLT compared to CVLT.

Supplementary Table S4. Effect of *TCN1* Expression on Memory Performance Stratified by Diagnosis

	CTRL		SCH		BD	
	std β	p-value	std β	p-value	std β	p-value
TCN1	-0.036	0.29	-0.10	0.039*	-0.15	0.011*
Age	-0.047	0.17	-0.13	0.0089*	-0.098	0.097
Sex (female)	0.11	0.0013*	0.19	1.16e-4*	0.15	0.012*

The standardized and combined CVLT and HVLT scores were used as the memory metric.
Std β: Standardized regression coefficients. * $p < 0.05$.

Supplementary Table S5. Number of Subjects Tested with CVLT and HVLT within Each Diagnostic Group

	**CVLT**	**HVLT**	SUM
**CTRL**	228	316	544
**SCH**	301	127	428
**BD**	253	25	278
SUM	782	468	1250

Supplementary Figure S5. Scatter Plot of the Relationship between Standardized *TCN1* Expression Levels and Standardized Memory Scores. The composite memory score (cvlt_hvlt_z) was produced by combining z-transformed CVLT and HVLT scores using both the discovery and replication samples.

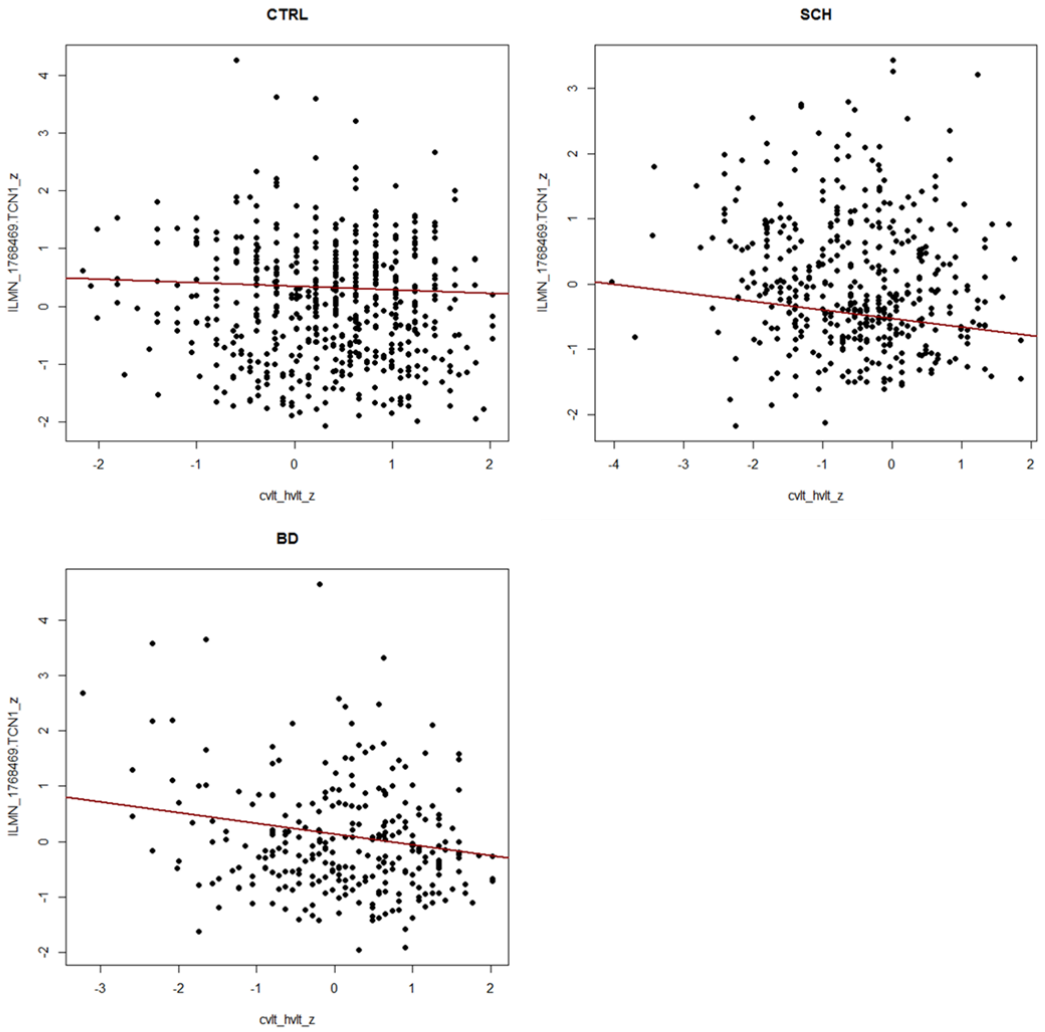

Supplementary Table S6. Demographic and Clinical Characteristics of Discovery Sample

	BD (n=234)		SCH (n=291)		CTRL (n=229)		Chi-square	
	n	%	n	%	n	%	X^2	p
Sex (female)	142	60.7	118	40.6	105	45.9	21.9	<.001
Ethnicity (European)	221	94.4	251	86.3	229	100	64.7	<.001

	BD		SCH		CTRL		ANOVA	
	mean	sd	mean	sd	mean	sd	*F*	p
Age (years)	34.8	12.0	31.4	10.0	32.7	9.7	6.7	<.01
PANSS total score	46.9	11.2	64.9	17.3			184.6	<.001
YMRS total score	3.7	5.0	5.4	4.9			14.2	<.001
IDS total score	18.1	12.7	18.8	12.7			0.4	.52
Cobalamin (pmol/L)	326.8	124.9	325.8	150.2	307.4	107.4	0.9	.41

748 of 754 individuals (99.2%) in the discovery sample had sufficient cobalamin levels. Cobalamin sufficiency was defined as >150 pmol/L (51). BD: Bipolar disorder. SCH: Schizophrenia. CTRL: Healthy controls. PANSS: Positive and Negative Symptom Scale. YMRS: Young Mania Rating Scale. IDS: Inventory of Depressive Symptoms.

Supplementary Table S7. Demographic and Clinical Characteristics of Replication Sample

	BD (n=67)		SCH (n=195)		CTRL (n=316)		Chi-square	
	n	%	n	%	n	%	X^2	p
Sex (female)	36	53.7	72	96.9	125	39.4	6.0	.048
Ethnicity (European)	52	77.6	150	76.9	311	98.1	78.5	<.001

	BD		SCH		CTRL		ANOVA	
	mean	sd	mean	sd	mean	sd	*F*	p
Age (years)	32.6	11.3	29.0	8.4	31.7	7.6	7.9	<.001
PANSS total score	43.2	8.9	61.7	15.5			85.5	<.001
YMRS total score	4.2	5.6	4.8	5.3			0.5	.48
IDS total score	14.1	9.9	13.8	10.7			0.03	.86
Cobalamin (pmol/L)	203.4	118.5	227.2	117.8	216.8	105.8	1.04	.35

419 of 578 individuals (72.5%) in the replication sample had sufficient cobalamin levels. Cobalamin sufficiency was defined as >150 pmol/L (51). BD: Bipolar disorder. SCH: Schizophrenia. CTRL: Healthy controls. PANSS: Positive and Negative Symptom Scale. YMRS: Young Mania Rating Scale. IDS: Inventory of Depressive Symptoms.

RNA microarray preprocessing and quality control

Multidimensional scaling and hierarchical clustering were used for regular quality control and removal of multiple batch effects (RNA extraction batch, RNA extraction method, DNase treatment batch, cRNA labelling batch, and chip hybridization). The "detectOutlier" function in the R package *lumi* was used to detect outlier samples based on distance to the cluster center (defined as the average of all samples after removing 10 percent of the samples farthest away from the center). This function detects a sample as outlier when its distance to the center is larger than a certain threshold (we used the default threshold of 2*median distance to the center). In general, the samples that were removed by this QC step had lower numbers of detected transcripts and low signal-noise ratio. In total, 84 samples were identified as outliers and removed from the data file. Since the reproducibility of Illumina gene expression chips is generally high, and since the technology implies that several beads on the array carry the same probe as a built-in technical replicate, we did not perform any separate analysis of technical replicates. The R package *illuminaHumanv4.db* was used to map Illumina probe identifiers to gene symbols. Mappings were based on data provided by Entrez Gene (ftp://ftp.ncbi.nlm.nih.gov/gene/DATA). Further, to make sure that the significantly associated probes were correctly annotated, we mapped the probe sequences provided by Illumina directly to the human reference using the BLAT tool available at the University of California Santa Cruz Genome Browser website (http://genome.ucsc.edu/).
